# Antiangiogenesis for Advanced Non-Small-Cell Lung Cancer in the Era of Immunotherapy and Personalized Medicine

**DOI:** 10.3389/fonc.2017.00052

**Published:** 2017-03-29

**Authors:** Samer Tabchi, Normand Blais

**Affiliations:** ^1^Hematology-Oncology Department, Centre Hospitalier de l’Université de Montréal, Montréal, QC, Canada

**Keywords:** antiangiogenesis, combination therapy, immunotherapy, non-small-cell lung cancer, angiogenesis

## Abstract

Over the past decade, patients with advanced non-small-cell lung cancer (NSCLC) have witnessed substantial advances in regards to therapeutic alternatives. Among newly developed agents, angiogenesis inhibitors were extensively tested in different settings and have produced some favorable outcomes despite several shortcomings. Bevacizumab is the most examined agent in this context and has demonstrated significant survival benefits when combined with standard chemotherapy in eligible patients. Preliminary results on the addition of bevacizumab to erlotinib in patients with EGFR-mutated NSCLC seem promising. Other antiangiogenic agents were also tested, but ramucirumab and nintedanib are the only agents with a positive impact on survival. More recently, immune checkpoint inhibitors (ICIs) have had considerable success due to their prolonged durations of response, yet response rates are still deemed suboptimal, and various combination therapies are being tested in an effort to improve efficacy. Preclinical evidence suggests an immunosuppressive effect of pro-angiogenic factors, which sets up a plausible rationale for combining ICIs and antiangiogenic agents. Herein, we review the landmark data supporting the success of angiogenesis inhibitors, and we discuss the potential for combination with immunotherapy and targeted agents.

## Introduction

A decade has now passed since bevacizumab, the first promising antiangiogenic agent, was approved for the treatment of non-small-cell lung cancer (NSCLC), and the lessons learned revealed that clinical applications of antiangiogenesis are somewhat more challenging than initially believed (1). As a fully humanized monoclonal antibody (mAb) that binds vascular endothelial growth factor-A (VEGF-A) and prevents interaction with VEGFR-1 and VEGFR-2 (the primary receptors involved in endothelial cell proliferation and migration), bevacizumab was thought of as a “silver bullet” capable of targeting multiple types of cancer since tumor proliferation and spread depend on neo-vasculature (2–4). However, despite survival gains attributed to this agent, clinical trial results did not fully meet with the expectations and management of patients with advanced NSCLC still requires significant improvements in order to clearly affect outcomes in this first ranking cancer in terms of cancer-related mortality (5). Nevertheless, angiogenesis remained an area of active research, and numerous agents have been tested. These agents bind VEGFR-2 directly (e.g., ramucirumab), act as VEGF inhibitors (e.g., aflibercept), or block intracellular downstream signal transduction by the inhibition of the tyrosine kinases of VEGF receptors (e.g., sorafenib and nintedanib) (6–8).

In the era of immunotherapy and refined precision medicine, the value of antiangiogenic agents and their cost-efficiency could be put into question in the face of more successful biologic agents such as immune checkpoint inhibitors (ICIs) that demonstrated significant clinical activity both in the first- and second-line setting with much promise attributed to the durable responses they achieve in responding patients (9). On the other hand, combining immunotherapy and angiogenesis inhibitors could prove to be a successful undertaking, which might improve the efficacy of both agents. Herein, we will provide a review of noteworthy data relating to successful antiangiogenic agents in NSCLC, be it in combination with chemotherapy or with newer agents.

## Targeting VEGF

### Bevacizumab

#### Combination with Cytotoxic Therapy

The initial randomized phase II study of this anti-VEGF-A mAb evaluated two different doses of bevacizumab (7.5 and 15 mg/kg) in addition to paclitaxel/carboplatin vs. chemotherapy alone, and the results demonstrated significant improvements in terms of response rate (RR) (31.5 vs. 18.8%) and median time to progression (7.4 vs. 4.2 months, *p* = 0.023) in favor of the arm with the highest dose of bevacizumab compared with the control arm (10). A noteworthy outcome of this trial was the identification of clinical features that were associated with high rates of life-threatening hemoptysis. Therefore, centrally located tumors with proximity to major blood vessels, cavitation, and squamous cell histology became exclusion criteria in most of the subsequent studies. However, ensuing data from the phase 4 SAiL study and the ARIES Observational Cohort study called into question whether cavitation and centrally located tumors did affect the rate of severe hemoptysis (11). Consequently, expert opinion suggests that squamous histology and the presence of hemoptysis are the most important contraindications to bevacizumab (12).

Following the success of the phase II study, a large phase III trial with a similar design conducted by the Eastern Cooperative Oncology Group (ECOG)—ECOG 4599—confirmed the benefits of bevacizumab (at a dose of 15 mg/kg), in the same setting, in terms of overall survival (OS) (12.3 vs. 10.3 months, *p* = 0.003), RR (35 vs. 15%, *p* < 0.001), and progression free survival (PFS) (6.2 vs. 4.5 months, *p* < 0.001) (13). In Europe, the AVAiL phase III trial also attempted to confirm the benefit of bevacizumab but in combination with the cisplatin/gemcitabine doublet and at two different dose levels (7.5 and 15 mg/kg) (14). Although the improvements in PFS were statistically significant for both dose levels of bevacizumab (6.5 vs. 6.1 months, *p* = 0.03 for the higher dose and 6.7 vs. 6.1, *p* = 0.003 for the lower dose), the study design did not allow for a direct comparison between both dose levels. Additionally, a subsequent survival analysis failed to demonstrate any OS benefit (15). Considering the modest absolute value of PFS improvements and the absence of any OS benefit, some experts favor the addition of bevacizumab to a paclitaxel/carboplatin regimen and support a theory that paclitaxel might be more susceptible to positive modulation by bevacizumab (16–18). The results of the BEYOND study are in line with this reasoning. This more recent phase III study, evaluating the addition of bevacizumab (15 mg/kg) to a carboplatin/paclitaxel backbone chemotherapy regimen in a Chinese cohort, demonstrated significant improvements in PFS [9.2 vs. 6.5 months; hazard ratio (HR), 0.40; 95% CI, 0.29–0.54; *p* < 0.001] and OS (24.3 vs. 17.7 months; HR, 0.68; 95% CI, 0.50–0.93; *p* = 0.0154) (19). Of particular note, the very favorable outcomes in terms of PFS and OS in the control arm seem to reflect a better selection of patients along with improvements in supportive care measures. Additionally, subsequent lines of therapy have most definitely impacted survival results in both arms as the EGFR mutation rates were 27 and 26% and the subsequent use of EGFR-TKI was 36 and 38% for the experimental and standard arms, respectively (Table 1) (19).

**Table 1:** **Results of landmark trials evaluating antiangiogenic agents in metastatic non-small-cell lung cancer**.

Study/phase	Chemotherapy	Number of patients (*n*)	ORR (%)	Median PFS (months)	HR (95% CI); *p*	Median OS (months)	HR (95% CI); *p*
ECOG 4599/phase III	Pac/Carbo	444	15	4.5	HR = 0.66 (0.57–0.77); *p* < 0.001	10.3	HR = 0.79 (0.67–0.92); *p* = 0.003
	Pac/Carbo/Bev	434	35	6.2		12.3	

AVAiL/phase III	Cis/Gem	345	21.6	6.1	–HR = 0.75 (0.64–0.87); *p* = 0.0003	13.1	–HR = 0.93 (0.78–1.11); *p* = 0.420
	Cis/Gem/Bev						
	–7.5 mg/kg	–345	–37.8	–6.7	–HR = 0.85 (0.73–1.00); *p* = 0.0456	–13.6	–HR = 1.03 (0.86–0.54); *p* < 0.01
	–15 mg/kg	–351	–34.6	–6.4		–13.4	

BEYOND/phase III	Pac/Carbo	138	26	6.5	HR = 0.40 (0.29–0.54); *p* < 0.01	17.7	HR = 0.68 (0.50–0.93); *p* = 0.0154
	Pac/Carbo/Bev	138	54	9.2		24.3	

AVAPERL/phase III	Cis/Pem/Bev	376	–	–	–	–	–
	Pem/Bev maintenance	128	–	7.4	HR = 0.57 (0.44–0.75); *p* < 0.0001	17.1	HR = 0.87 (0.63–1.21); *p* = 0.29
	Bev maintenance	125	–	3.7		13.2	

POINTBREAK/phase III	Carbo/Pem/Bev → Bev/Pem maintenance	472	34	6	HR = 0.83 (0.71–0.96); *p* = 0.012	13.4	HR = 1.0 (0.86–1.16); *p* = 0.949
	Carbo/Pac/Bev → Bev maintenance	467	33	5.6		12.6	

PRONOUNCE/phase III	Carbo/Pem → Pem	182	23.6	4.44	HR = 1.06 (0.84–1.35); *p* = 0.610	10.5	HR = 1.07 (0.83–1.36); *p* = 0.615
	Carbo/Pac/Bev → Bev	179	27.4	5.49		11.7	

JO25567/phase II	Erlotinib	75	64	9.7	HR = 0.54 (0.36–0.79); *p* = 0.0015	–	–
	Erlotinib/Bev	77	69	16.0		–	

BELIEF/phase II	Erlotinib/Bev	109	–	13.6	–	–	–
	All patients	60	70.3	15.4		–	
	T790M-mutated EGFR						

REVEL^a^/phase III	Docetaxel	625	14	3.0	HR = 0.76 (0.68–0.86); *p* < 0.0001	9.1	HR = 0.86 (0.75–0.98); *p* = 0.023
	Docetaxel/ramucirumab	628	23	4.5		10.5	

LUME-lung 1^a^/phase III	Docetaxel	659	1.5	1.5	HR = 0.63 (0.48–0.83); *p* = 0.0008	9.1	HR = 0.94 (0.83–1.05); *p* = 0.2720
	Docetaxel/nintedanib	655	4.9	3.6		10.1	

LUME-lung 2^a^/phase III	Pem	360	8.3	3.6	HR = 0.83 (0.70–0.99); *p* = 0.0435	12.7	HR = 1.03 (0.85–1.21); *p* = 0.8940
	Pem/nintedanib	353	9.1	4.4		12.2	

*HR, hazard ratio; PFS, progression free survival; OS, overall survival; Pac, paclitaxel; Carbo, carboplatin; Bev, bevacizumab; Cis, cisplatin; Gem, gemcitabine; Pem, pemetrexed; ECOG, Eastern Cooperative Oncology Group*.

*^a^Trials in the second-line setting*.

To date, the available data were compiled in two different meta-analyses of platinum doublets combined with bevacizumab and both concluded to significant PFS and RR benefit from the addition of bevacizumab to standard cytotoxic therapy (20, 21). However, only one of these studies demonstrated a 10% relative reduction in the risk of death with the addition of bevacizumab to chemotherapy (HR: 0.90, 95% CI, 0.81–0.99) (21).

Bevacizumab was also tested in the adjuvant setting at a dose of 15 mg/kg in combination with cisplatin and vinorelbine, docetaxel, gemcitabine, or pemetrexed (for non-squamous histology) per physician’s choice. The results of the E1505 phase III study were released after an interim analysis showed futility 41 months of follow-up. Additionally, patients receiving bevacizumab-containing therapy had significantly higher rates of grade 3–5 toxicities, mostly in the form of hypertension (8 vs. 30%), neutropenia (33 vs. 38%), and overall worst grade (67 vs. 84%) (22).

#### Maintenance Therapy and Dosing

Besides the issue of optimal backbone chemotherapy, other pivotal questions involve the duration of therapy with bevacizumab and the optimal dose of this agent.

In the landmark ECOG 4599 study, bevacizumab was continued until progression or limiting toxicities, and a retrospective analysis demonstrated superior PFS and OS in patients where bevacizumab was continued (PFS: 4.4 vs. 2.8; HR: 0.64, and OS: 12.8 vs. 11.4; HR: 0.75) (13, 23). Since then, three important studies have addressed this issue. The POINTBREAK trial did not demonstrate any OS advantage when pemetrexed/carboplatin/bevacizumab (15 mg/kg) followed by bevacizumab/pemetrexed maintenance was compared with paclitaxel/carboplatin/bevacizumab followed by bevacizumab maintenance, but PFS favored the pemetrexed containing regimen (6.0 vs. 5.6 months; *p* = 0.012). The AVAPERL study comparing cisplatin/pemetrexed/bevacizumab followed by either pemetrexed or pemetrexed/bevacizumab maintenance, in non-progressing patients, demonstrated a substantial PFS advantage in favor of the doublet maintenance (7.4 vs. 3.7 months; HR, 0.57; 95% CI, 0.44–0.75; *p* < 0.0001), but OS did not reach statistical significance. The PRONOUNCE study did not demonstrate a survival difference when pemetrexed/carboplatin followed by pemetrexed maintenance was compared with paclitaxel/carboplatin/bevacizumab (15 mg/kg) followed by bevacizumab maintenance (Table 1) (24–26). When all these trials are taken together, it remains unclear whether the demonstrated benefit of maintenance pemetrexed is improved by bevacizumab. An ongoing phase III study with three different maintenance therapies (ECOG 5508; pemetrexed vs. bevacizumab vs. pemetrexed/bevacizumab) will provide further data in that regard.

Different doses of bevacizumab were tested in different settings, and in NSCLC both the higher (15 mg/kg every 3 weeks) and lower (7.5 mg/kg every 3 weeks) doses were tested, but direct comparison of both dose levels for efficacy was not performed in the larger landmark trials. However, the ABIGAIL trial, designed as a correlative biomarker finding study of bevacizumab combined to a platinum doublet, randomized patients to receive 7.5 or 15 mg/kg. Although survival was not the primary endpoint of this study and with the caveat of an insufficient patient cohort (*n* = 303) to adequately compare the clinical effect of dose, no difference in PFS and OS was observed between both dose levels of bevacizumab (27). Considering these data and results from the aforementioned meta-analyses suggesting similar clinical benefit from bevacizumab at both dose levels, the optimal dose of bevacizumab is still debatable (20, 21).

#### Combination with Tyrosine Kinase Inhibitors

The addition of bevacizumab to erlotinib was initially attempted in patients with refractory NSCLC who were unselected for activating EGFR mutations, but no improvements in survival were obtained with the combination therapy (28). More recently, a Japanese phase II study evaluated the same combination of erlotinib/bevacizumab in patients with treatment-naïve EGFR-mutated (exon 19 and 21 alterations) NSCLC (29). The results demonstrated a substantial improvement in PFS (16.0 vs. 9.7 months, HR 0.54, *p* = 0.0015), but the study was not powered to compare OS (29).

These encouraging results have been suggested to be due to an increased uptake of erlotinib in tumor cells that is potentiated by bevacizumab in addition to the actual blockade of angiogenic signaling (30).

The preliminary results of another open label single arm phase II trial from Europe, the BELIEF study, yielded provocative results and met its 1-year PFS endpoint for the entire cohort [55.6% (95% CI: 44.7–66.6%); median: 13.6 months], including patients with T790M-mutated NSCLC [1-year PFS: 60.2% (95% CI: 45.6–74.8%); median: 15.4 months] (31). Based on these results, erlotinib/bevacizumab received approval as first-line treatment of patients with EGFR-mutated NSCLC in June 2016 in Europe. Another ongoing study, the ACCRU (NCT01532089) trial, has completed accrual in the US, and its results will help confirm the available data.

## Targeting VEGF-R

### Ramucirumab

This fully human mAb targeting VEGFR-2 first demonstrated its efficacy in gastric and colorectal cancers (32–34). The development in NSCLC was somewhat more challenging. After the initial open label phase II data demonstrated favorable responses, another phase II study randomized patients to cisplatin/pemetrexed followed by pemetrexed maintenance vs. cisplatin/pemetrexed/ramucirumab followed by ramucirumab-pemetrexed maintenance (35, 36). Unfortunately, the latter trial did not meet its primary endpoint (PFS: 7.2 vs. 5.6 for the ramucirumab arm; *p* = 0.132) (36). Further development of ramucirumab in the first-line setting was subsequently halted.

The activity of ramucirumab in NSCLC was nonetheless demonstrated in the phase III REVEL trial, where a docetaxel/ramucirumab combination was compared to docetaxel alone in the second-line setting (Table 1) (37). Of note, patients who previously received bevacizumab and those who had squamous histology were not excluded. Modest but statistically significant improvements in OS [10.5 vs. 9.1 months; HR: 0.86 (0.75–0.98); *p* = 0.023] and PFS [4.5 vs. 3.0 months; HR: 0.76 (0.68–0.86); *p* < 0.0001] led to FDA approval in combination with docetaxel regardless of histological subtype. However, the use of ramucirumab is not widely adopted since some experts believe that the OS improvement, although statistically significant, might not be clinically meaningful in accordance with the ASCO definition for expensive drugs, particularly if these improvements come at the expense of added toxicities (38).

## Tyrosine Kinase Inhibitors of Angiogenesis

The appeal of antiangiogenic TKIs stemmed from their success in renal cell carcinoma as well as from their ease of administration, which led to further development in different other cancer types. Unfortunately, different TKIs failed to produce consistent success in the treatment of advanced NSCLC.

Combining sorafenib with a platinum doublet in the first-line setting did not demonstrate any survival benefit (39). Two studies evaluating sunitinib combined with erlotinib in the second-line setting in patients with wild-type EGFR, or with pemetrexed, also failed to demonstrate efficacy of the combination therapies (40, 41). Combining pazopanib with a platinum doublet resulted in excessive toxicities (42).

Among newer multi-kinase inhibitors, the phase II/III study evaluating cediranib in addition to frontline carboplatin/paclitaxel was halted for futility on the basis of excessive toxicities, and the phase III MONET trial testing motesanib, also in combination with frontline carboplatin/paclitaxel, did not result in significant OS improvements (43, 44). Another TKI, vandetanib, was assessed in four phase III trials, two of which (ZEAL and ZODIAC trials) evaluated the agent in combination with docetaxel or pemetrexed maintenance, whereas the other two studies tested vandetanib as a single agent in second or subsequent lines of therapy, but neither of these studies had a positive impact on survival, and the combination therapies mostly resulted in increased toxicities (45–48).

### Nintedanib

The triple angiokinase inhibitor nintedanib is the only TKI agent that has shown significant results when tested in the phase III LUME-Lung1 study (Table 1). This agent was tested in the second-line setting of patients with advanced NSCLC (both squamous and non-squamous histologies were included) in combination with docetaxel, and the study met its primary PFS endpoint in comparison with docetaxel monotherapy (3.4 vs. 2.7 months; HR, 0.79; 95% CI, 0.68–0.92; *p* = 0.0019) but failed to demonstrate differences in survival for the global population (49). When patients were evaluated in a prespecified subgroup analysis, the combination therapy showed improvements in OS for patients with an adenocarcinoma histology who progressed within 9 months of first-line therapy (10.9 vs. 7.9 months; HR, 0.75; 95% CI, 0.60–0.92; *p* = 0.0073) and for all patients with adenocarcinoma (12.6 vs. 10.3 months; HR, 0.83; 95% CI, 0.70–0.99; *p* = 0.0359). A confirmatory phase III trial, the LUME Columbus study (NCT02231164), with the same design, but excluding patients with squamous histology, was terminated for slow accrual. The LUME-Lung 2 study, examining a pemetrexed/nintedanib combination in the second-line setting, also demonstrated a modest but significant PFS improvement in comparison with pemetrexed monotherapy (PFS: 4.4 vs. 3.6 months; HR, 0.83, 95% CI, 0.70–0.99; *p* = 0.0435) but failed to demonstrate a survival benefit (Table 1) (50). As such, this agent has received approval in Europe for the second-line treatment of NSCLC in combination with docetaxel, but FDA approval has not been granted.

## Combinations with Immunotherapy

The demonstration of durable responses in patients with advanced NSCLC, through the use of ICIs, has led to considerable enthusiasm within the scientific community. The first anti-programmed death-1 (PD-1) agents, nivolumab and pembrolizumab, gained accelerated approval for the treatment of metastatic NSCLC in the second-line setting after demonstrating significant clinical activity in this context (51–53). Additionally, agents targeting programmed death-ligand 1 (PD-L1)—such as atezolizumab, durvalumab, and avelumab—are also in advanced stages of development, and some have gained approval in several other indications (54–56). Most recently, pembrolizumab was also found to be superior to standard platinum-based chemotherapy and gained approval for the first-line treatment of metastatic NSCLC with positive PD-L1 expression—defined as tumor proportion score of 50% or more (57). Despite their efficacy, reported overall RRs are less than optimal (20–25% in the second-line and 45% in the frontline setting for selected patients), which gives rise to different strategies aimed at improving responses to ICIs. Therefore, investigators are attempting combinations of ICIs with chemotherapy, radiation therapy, cancer vaccines, oncolytic viruses, and targeted therapies in order to overcome resistance mechanisms (58). Some evidence suggests that angiogenesis might be associated with immunosuppression within the tumor microenvironment thereby potentiating immune-escape of tumor cells (59).

The complex relationship between VEGF and tumor-related immune regulation involves several key pathways that lead to an immunosuppressive microenvironment. VEGF is effectively capable of inducing inhibitory immune cells such as T-regulatory cells (Tregs) and myeloid-derived suppressor cells (MDSCs) (60, 61). Additionally, exposure to VEGF at pathologic levels might inhibit the differentiation and/or emigration T-cell progenitors from the thymus resulting in a state of systemic cancer-related immunosuppression (62). Moreover, it seems that lymphocyte influx across the vascular endothelium toward the tumor is affected by VEGF, which leads to a defect in intercellular adhesion molecule-1 and vascular cell adhesion molecule-1 clustering at the endothelial cell surface through nitric oxide production and subsequently leads to defective lymphocyte adhesion and migration toward the tumor environment (63).

On the other hand, preclinical models have shown that the use of antiangiogenic agents such a sunitinib or cabozantinib lead to an increase in CD4+ and CD8+ T-cells infiltration and reduce PD-1 expression within these cells while the influx of MDSCs and Tregs toward tumor tissue seems to be decreased (64–66).

In light of these findings, multiple trials are currently investigating combinations of immunotherapy and antiangiogenic drugs in different types of cancer. The most encouraging results in this context come from the experience with melanoma, where immunotherapy achieved its first successes. Promising phase I data indicated that a combination of ipilimumab and bevacizumab is both safe and effective with a median OS of 25.1 months and a disease control rate of 67.4%, thereby supporting the preclinical rationale of VEGF impact on immune regulation (67).

In NSCLC, preliminary data from a phase I study evaluating a nivolumab/bevacizumab combination vs. nivolumab monotherapy as maintenance after initial platinum-based chemotherapy suggested a favorable adverse-events profile for both arms (68). This study is certainly not powered to provide information in regards to optimal regimens, but the results indicated median PFS values with combination therapy that compared favorably to those obtained with the comparator arm (PFS: 37.1 weeks for nivolumab/bevacizumab, whereas nivolumab monotherapy yielded 16 and 21.4 weeks of PFS in patients with squamous and non-squamous histology, respectively) (68).

Another phase Ia/dose-limiting toxicity evaluation explored the addition of ramucirumab to pembrolizumab in patients with advanced NSCLC, gastric-esophageal cancers, and urothelial carcinoma (69). Preliminary data also indicate the safety of this combination as no dose-limiting toxicities were identified in patients with NSCLC (only one patient with urothelial carcinoma experienced severe toxicities requiring treatment discontinuation).

These encouraging safety data will certainly need to be cemented with efficacy data from larger trials exploring ICIs/antiangiogenesis combinations before any definitive conclusions can be drawn. Several challenges involving optimal dosing and treatment schedules remain to be resolved before such combinations can be considered for clinical practice especially since several combinations involving immunotherapy (with chemotherapy, radiation therapy, vaccines, etc.) are being tested and could have better efficacy when tested in larger trials.

## Conclusion

Identifying the VEGF pathway as a key regulator in angiogenesis and in subsequent tumor growth and metastasis has led to the development of several agents targeting the pathway’s different components. Bevacizumab appears to be the most successful antiangiogenic, but ramucirumab and nintedanib have also demonstrated clinical efficacy in the second-line setting. Although some experts believe that the benefits of these agents have plateaued, the promising results of an erlotinib/bevacizumab combination in EGFR-mutated lung cancer have proven otherwise. The intricate relationship between immunosuppression and angiogenesis indicates that a synergistic relationship could result from a combination of ICIs and angiogenesis inhibitors with relatively favorable toxicity profiles and has sparked a renewed interest in the study of antiangiogenic drugs. However, our comprehension of cancer-related immune modulation barely scratches at the surface of a vast compendium of knowledge. Many challenges need to be addressed before optimal combination therapies are defined.

## Author Contributions

ST and NB contributed equally to the production of this manuscript.

## Conflict of Interest Statement

The authors declare that the research was conducted in the absence of any commercial or financial relationships that could be construed as a potential conflict of interest. The handling editor declared a past coauthorship with one of the authors (NB) and states that the process nevertheless met the standards of a fair and objective review.
